# Nanopore-based targeted next-generation sequencing of tissue samples for tuberculosis diagnosis

**DOI:** 10.3389/fmicb.2024.1403619

**Published:** 2024-07-03

**Authors:** Weiwei Gao, Chen Yang, Tianzhen Wang, Yicheng Guo, Yi Zeng

**Affiliations:** Department of Tuberculosis, The Second Hospital of Nanjing, Nanjing, China

**Keywords:** extrapulmonary tuberculosis, tissue sample, targeted next-generation sequencing (tNGS), TB diagnosis, drugresistant TB, *Mycobacterium tuberculosis*, hematoxylin and eosin staining

## Abstract

**Objective:**

Diagnosing tuberculosis (TB) can be particularly challenging in the absence of sputum for pulmonary tuberculosis cases and extrapulmonary TB (EPTB). This study evaluated the utility of nanopore-based targeted next-generation sequencing (tNGS) for diagnosing TB in tissue samples, and compared its efficacy with other established diagnostic methods.

**Methods:**

A total of 110 tissue samples from clinical cases were examined. The sensitivity and specificity of tNGS were benchmarked against a range of existing diagnostic approaches including hematoxylin and eosin (HE) staining in conjunction with acid-fast bacilli (AFB) detection, HE staining combined with PCR, HE staining paired with immunohistochemistry (IHC) using anti-MPT64, and the Xpert *Mycobacterium tuberculosis* (MTB)/rifampicin (RIF) assay.

**Results:**

The sensitivity and specificity of tNGS were 88.2 and 94.1%, respectively. The respective sensitivities for HE staining combined with AFB, HE staining combined with PCR, HE staining combined with IHC using anti-MPT64, and Xpert MTB/RIF were 30.1, 49.5, 47.3, and 59.1%. The specificities for these methods were 82.4, 88.2, 94.1, and 94.1%, respectively. Analysis of drug resistance based on tNGS results indicated that 10 of 93 TB patients (10.75%) had potential drug resistance.

**Conclusion:**

Targeted next-generation sequencing achieved higher accuracy than other established diagnostic methods, and can play a crucial role in the rapid and accurate diagnosis of TB, including drug-resistant TB.

## 1 Introduction

Tuberculosis (TB) is a preventable and curable disease (Wu et al., 2020). However, in 2023, it was the second leading cause of death from a single infectious agent worldwide, causing almost twice as many deaths as HIV/AIDS. More than 10 million people continue to fall ill with tuberculosis every year (World Health Organization, 2011). The pathogen, *Mycobacterium tuberculosis* (MTB), can affect the lungs (pulmonary TB, PTB) and other organs (extrapulmonary TB, EPTB).

Timely diagnosis is paramount for effective management of TB. Traditional diagnostic methods, such as smear and culture tests, often fail to detect PTB in the absence of sputum (Silva et al., 2019), and EPTB with a low bacterial load (Chakravorty et al., 2005). In recent years, tissue biopsy has been increasingly employed for rapid and accurate diagnosis of smear-negative TB and EPTB (Lin et al., 2022; Barker et al., 2023). Current tissue biopsy methods for TB diagnosis include staining techniques such as hematoxylin and eosin (HE) combined with acid-fast staining (AFB), HE combined with PCR, HE combined with immunohistochemistry (IHC) using anti-MPT64, and the Xpert MTB/rifampicin (RIF) assay. However, HE staining lacks specificity and frequently reveals granulomatous inflammation with or without necrosis (Rui-e, 2020). Although AFB staining is a straightforward method for screening mycobacteria, it exhibits low sensitivity and can struggle to distinguish non-TB mycobacteria (NTM) (Jain et al., 2017) and certain leprosy cases (Ghosh et al., 2017). The GeneXpert MTB/RIF (Xpert) assay, recommended by the World Health Organization, has been widely used in clinical practice (World Health Organization, 2023). Although this assay is universally accessible in most clinical settings for identifying RIF resistance, it does not meet the clinical requirements for testing resistance of other anti-TB drugs (Chen and Shen-jie, 2023). Although PCR has potential for diagnosing TB, its sensitivity is curtailed by issues such as non-specific amplification and base mismatch (Callahan et al., 2019), compromising the diagnostic utility of puncture tissues in TB detection (Raveendran and Wattal, 2016). Adoption of IHC for TB as a standard diagnostic technique in histopathology laboratories has been relatively sluggish, likely attributed to the absence of a universally applicable anti-mycobacterial antibody that can be used for all tissue types (Kohli et al., 2014).

Previous studies have demonstrated the high sensitivity and specificity of targeted next-generation sequencing (tNGS) in diagnosing pulmonary TB (Kambli et al., 2021) and NTM strains (Chen et al., 2023), as well as its efficacy in identifying drug-resistant TB (Cabibbe et al., 2020; de Araujo et al., 2023; Mansoor et al., 2023). However, further investigation is needed to determine the performance of tNGS for histopathology samples. To validate the use of tNGS and establish its practicality in the diagnosis of TB using tissue samples, a retrospective study was conducted that compared the outcomes of tNGS with those of HE staining combined with AFB, HE staining paired with PCR, HE staining coupled to IHC using anti-MPT64, and the Xpert MTB/RIF assay.

## 2 Materials and methods

### 2.1 Participants

**Figure 1:**
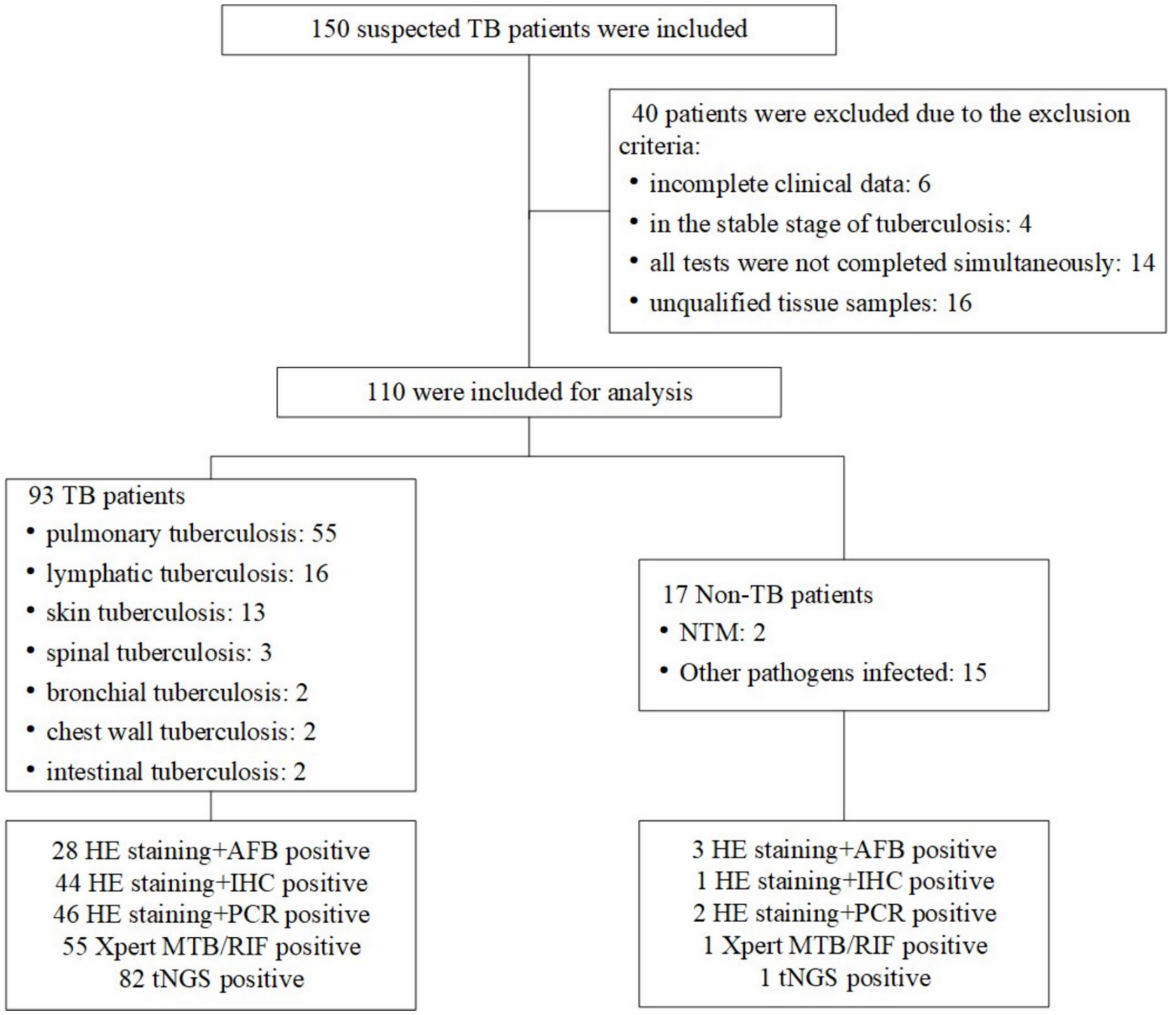
Flowchart showing the classification of patients included in the study.

A total of 150 patients suspected of TB were consecutively included in the study at the Second Hospital of Nanjing, China, between January 2021 and October 2023. These patients were sufficiently representative of all TB patients at the hospital. Diagnosis of TB was based on clinical characteristics, microbiological smear and culture, histopathology, cytology, radiology, and response to anti-tubercular therapy. Active TB was diagnosed for 110 patients and all were included in the study. All ultimate clinical diagnoses relied on the two most authoritative health industry standards in China: Diagnosis for pulmonary tuberculosis (WS288-2017) and Classification of tuberculosis (WS196-2017). The results of all diagnostic tests for all patients were compared independently with those of these gold standard techniques (Figure 1). These participants exhibited typical symptoms associated with the site of EPTB; the most common symptoms are fever (36.56%), cough or expectoration (20.43%) and night-sweat (12.90%). The results of the erythrocyte sedimentation rate and C-reactive protein demonstrated a possible inflammation reaction (Table 1).

**Table 1:** Clinical characteristics of the included patients.

Characteristics	All (*n* = 110)	TB (*n* = 93)	Non-TB (*n* = 17)
Age (year, mean ± SD)	46.0 ± 18.7	45.0 ± 17.3	48.7 ± 20.0
Male (*n*, %)	53 (48.2)	45 (48.4)	8 (47.1)
Female (*n*, %)	57 (51.8)	48 (52.6)	9 (52.9)
BMI (kg/m^2^)	21.0 ± 3.5	21.0 ± 3.3	19.5 ± 3.1
Symptoms			
Fever	37 (33.64)	34 (36.56)	3 (17.65)
Cough or expectoration	22 (22.00)	19 (20.43)	3 (17.65)
Night-sweat	20 (18.18)	12 (12.90)	6 (35.29)
Chest and back pain	12 (10.91)	8 (8.60)	0
Physical examination	23 (20.91)	22 (23.66)	2 (11.76)
Other symptoms	29 (23.36)	27 (29.03)	3 (17.65)
Laboratory examination			
Erythrocyte sedimentation rate (mm/h)	53.00 (26.00,88.00)	63.00 (22.00,97.00)	42.00 (26.00,58.00)
C-reactive protein (mg/L)	64.00 (26.34,107.32)	45.32 (17.82,92,16)	66.00 (12.30,77.25)

### 2.2 Procedures

Tissue samples were obtained through puncture, biopsy, and surgical resection. All specimens underwent various staining techniques including HE staining combined with AFB, HE staining combined with PCR, HE staining combined with IHC using anti-MPT64, Xpert MTB/RIF assay, and tNGS. All specific experimental processes are described below.

#### 2.2.1 HE staining combined with AFB

Tissue specimens were deparaffinized and rinsed with consecutive dilutions of alcohol. After heat fixation, specimens were washed with carbol fuchsin and incubated with HCl. Brilliant Green was used for counterstaining. After rinsing, samples were allowed to dry at room temperature. A positive result was considered positive even if the patient underwent multiple AFB microscopy examinations.

#### 2.2.2 HE staining combined with PCR

The MPB64 gene is widely recognized as a gene target for PCR diagnosis due to its high specificity in detecting extrapulmonary tuberculosis (Chaudhari et al., 2021). Therefore, in this study, DNA was extracted from tissue homogenates and a PCR assay targeting the MPB64 gene was conducted. DNA amplification was performed in the total reaction volume. The amplified product was electrophoresed and visualized after cycles of denaturation, annealing, and extension. A positive test was indicated by the presence of a 240 bp fragment.

#### 2.2.3 HE staining combined with IHC

For IHC staining, 3–4 micron sections were cut from the tissue block and incubated overnight. After deparaffinization, hydration, and microwave antigen retrieval, the endogenous peroxidase activity was inhibited. Subsequently, primary antibody (anti-BCG) and secondary antibody (anti-MPT64) were added. After staining with chromogen, hematoxylin was used as the contrast dye. Each staining included a negative and positive control.

#### 2.2.4 Xpert MTB/RIF assay

The GeneXpert MTB/RIF kits utilized in this study were sourced from Cepheid (City, State, United States). Firstly, the specimen was mixed with sample treatment reagent and incubated for 15 min. Subsequently, the mixture was transferred into a GeneXpert cartridge using the sterile dropper provided with the kit, and then loaded into the GeneXpert machine. Within 2 h, the GeneXpert system automatically displayed the results.

#### 2.2.5 Targeted next-generation sequencing

For sample processing, samples were digested with proteinase K and lysozyme, ground by zirconia grinding beads for 1 min, and lysozyme solution was added. The resulting lysate was used for subsequent nucleic acid extraction with a QIAamp DNA microbiome kit (Qiagen, City, Canada). Using the non-template control (NTC) method, blank EB buffer was used as a negative control for extracted nucleic acids. The concentration of extracted DNA was measured using a Qubit dsDNA quantification assay kit (Thermo Fisher, City, State, United States). Subsequently, the bacterial 16S rRNA gene was detected using universal primer pair 27F/1492R, and the fungal IST1/2 gene was amplified by PCR using primer pair ITS1/4. PCR amplification was performed using an ABI 2720 thermocycler with an initial denaturation step at 95°C for 3 min, followed by cycles at 95°C for 30 s, 62°C for 60 s, and 72°C for 60 s, and a final extension at 72°C for 3 min. PCR amplification products were purified and quantified using Invitrogen Qubit 4 for subsequent library preparation and TNPseq sequencing.

Nanopore barcode PCR was performed on the aforementioned products according to the instructions supplied with the PCR Barcode Expansion Pack 1–96 (EXP-PBC096) to construct libraries. After chip start-up, ~100 ng library pools were loaded into the nanopore flow cell and sequenced using the GridION platform. MinKNOW version 2.0 software was used to output data for base conditioning, and barcode demultiplexing was performed using Porechop.

Reads of small size (length < 200 bp) were filtered out using NanoFilt, and the remaining high-quality reads were aligned to all targets and potential etiologies using the National Center for Biotechnology Information (NCBI) Basic Local Alignment Search Tool (BLAST). Pathogens were categorized at the species level based on coverage and identity after NTC filtering. In general, the top 10 microorganisms sorted by aligned reads with a relative abundance score > 0.5% were classified as pathogens and further evaluated. MTB was considered positive when at least one sequence was mapped to a species or genus. All these steps in the TNPseq assay were conducted according to a previous report (Liu et al., 2024) with modifications.

### 2.3 Statistical analysis

In this study, we used R version 4.3.2 for statistical analysis. We calculated the sensitivity, specificity, positive predictive value (PPV), negative predictive value (NPV), and AUC value for the five detection methods using the measured TP, FP, FN, and TN values to evaluate the diagnostic effectiveness of tNGS compared with the other detection methods. Additionally, we used the Chi-square test to compare differences in positive rates for pathogenic identification between tNGS and the other detection methods. Furthermore, we compared the etiological positive rates of tNGS and the other detection methods for the three main biopsy tissues using the Chi-square test or Fisher’s exact probability method. Additionally, we created Venn and upset diagrams to illustrate the independent positive detection capability and applicability for the joint detection for TB. Finally, a scatter plot was used to demonstrate the diagnostic value of tNGS for clinical specimens with low bacterial load (especially in cases of EPTB).

### 2.4 Ethics approval

The study was approved by the Ethical and Institutional Review Boards for Human Investigation of the Second Hospital of Nanjing (ID: 2024-LS-ky026). Written informed consent was obtained from the patients themselves.

## 3 Results

### 3.1 Overview of clinical specimens from various tissues and detection of TB using tNGS

**Figure 2:**
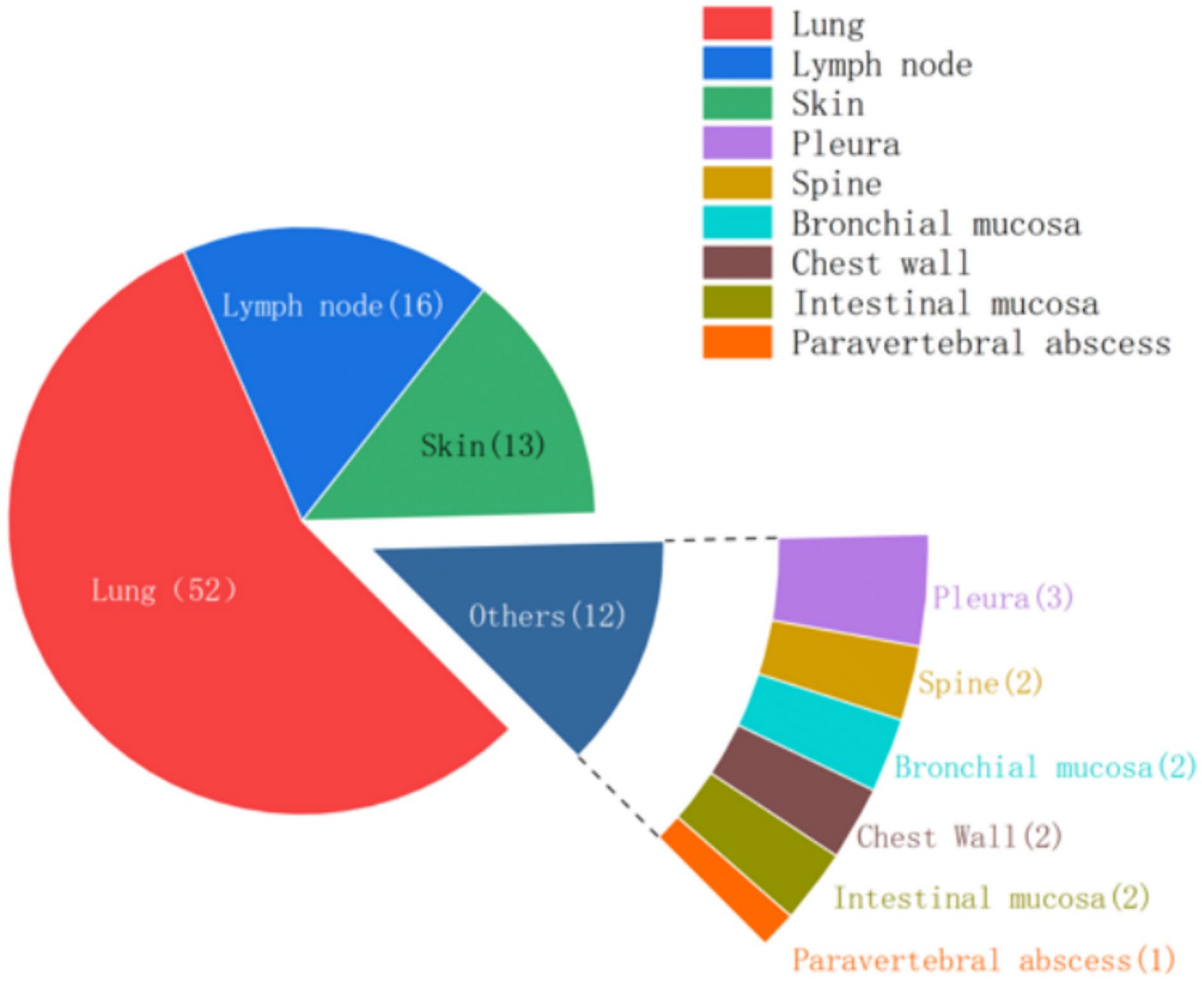
Fan chart of positive clinical specimens from various tissues.

**Figure 3:**
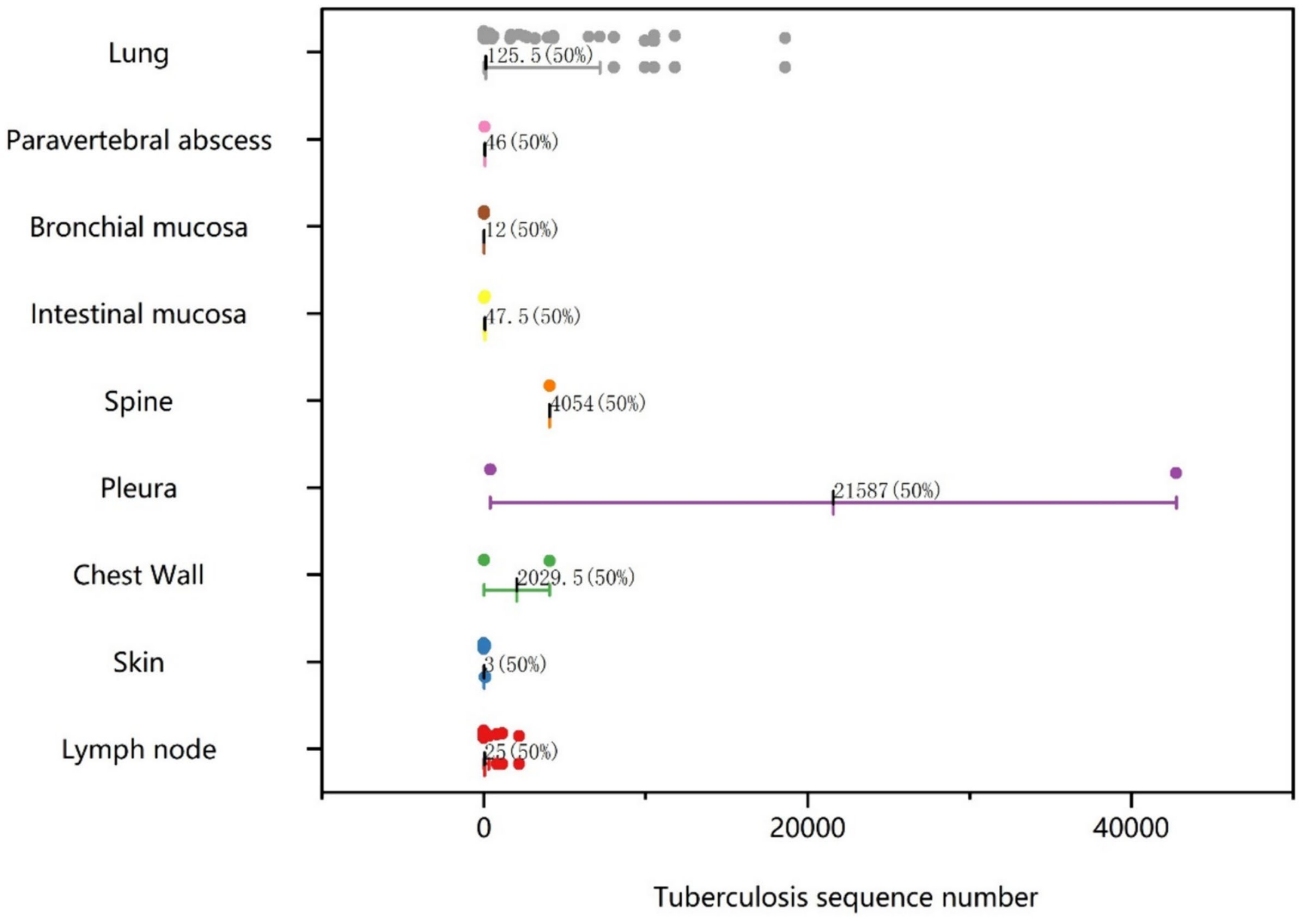
Scatter plot of TB sequence numbers for tissue samples testing positive by tNGS.

Histological samples spanned 10 distinct tissues: intestinal mucosa, lung, abdominal wall, spine, lymph node, skin, chest wall, pleura, bronchial mucosa, and paravertebral abscess. As depicted in Figure 2, positive results for TB were obtained from histological samples of all tissues except pleura. The results confirmed the efficacy of tNGS in identifying TB across diverse samples. Notably, 48 of 52 lung specimens and three pleural specimens were positive for PTB, with only seven cases undetected. For lymph node samples, 15 of 16 cases were identified, while 11 of 13 cases were detected for skin samples. In the case of spinal and paravertebral abscess specimens, two of three cases were identified. All cases of bronchial, chest wall, and intestinal TB were successfully detected. Additionally, scatter plots were used to display the number of nucleic acid sequences detected by tNGS in various TB-positive specimens (Figure 3). The median TB sequences of histological specimens of lung, paravertebral abscess, bronchial mucosa, intestinal mucosa, spine, pleura, chest wall, and lymph node were 125.5, 46, 12, 47.5, 4,054, 21,587, 2029.5, 3, and 25, respectively. These results revealed that the majority of histological samples contained few bacteria, demonstrating the utility of tNGS as a potent diagnostic tool for samples with low bacterial load.

### 3.2 Comparison of sensitivity, specificity, PPV, and NPV for the five detection methods

**Figure 4:**
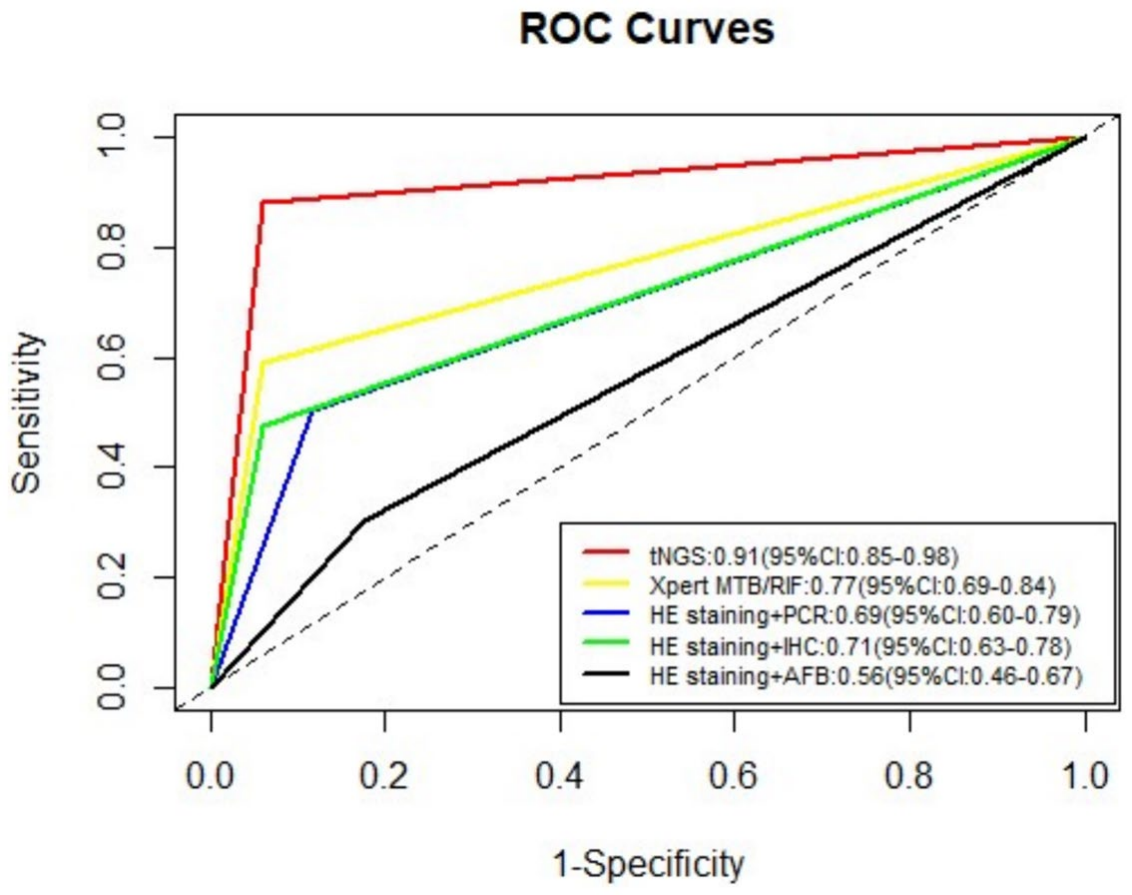
ROC curves for the five tests employed the diagnosis of TB.

Table 2 lists the sensitivity, specificity, PPV, and NPV values for the five detection methods employed in the diagnosis of TB. The tNGS method exhibited a sensitivity of 88.2% and a specificity of 94.1%. The sensitivity and specificity values for HE staining combined with AFB were 30.1 and 82.4%; for HE staining combined with PCR they were 49.5 and 88.2%; for HE staining combined with IHC with anti-MPT64 they were 47.3 and 94.1%; and for the Xpert MTB/RIF assay, they were 59.1 and 94.1%, respectively. Across all test methods, tNGS yielded the highest sensitivity and specificity. The positive likelihood ratios for tNGS, Xpert MTB/RIF, HE staining combined with PCR, HE staining paired with IHC, and HE staining coupled with AFB were 14.99 (95% confidence interval [CI], 2.24–100.51), 10.05 (95% CI, 1.49–67.82), 4.20 (95% CI, 1.13–15.71), 8.04 (95% CI, 1.19–54.51), and 1.71 (95% CI, 0.58–4.99), respectively. Receiver operating characteristic (ROC) curves for the five tests are displayed in Figure 4. The area under the curve (AUC) for tNGS in diagnosing TB was 0.91 (95% CI, 0.85–0.98), while that of Xpert MTB/RIF, HE staining combined with PCR, HE staining paired with IHC, and HE staining coupled with AFB was 0.77 (95% CI, 0.69–0.84), 0.69 (95% CI, 0.60–0.79), 0.71 (95% CI, 0.63–0.78), and 0.56 (95% CI, 0.46–0.67), respectively.

**Table 2:** Diagnostic efficiency of the five tests for the diagnosis of TB.

Test	Sensitivity (%)	Specificity (%)	PPV (%)	NPV (%)	Positive likelihood ratio
tNGS	**88.2** (95%CI:79.4–93.7)	**94.1** (95%CI:69.2–99.7)	**98.8** (95%CI:92.5–99.9)	**59.3** (95%CI:39.0–77.0)	14.99 (95%CI:2.24–100.51)
Xpert MTB/RIF	**59.1** (95%CI:48.4–69.1)	**94.1** (95%CI:69.2–99.7)	**98.2** (95%CI:89.2–99.9)	**29.6** (95%CI:18.4–43.8)	10.05 (95%CI:1.49–67.82)
HE staining + PCR	**49.5** (95%CI:39.0–60.0)	**88.2** (95%CI:49.8–92.2)	**95.8** (95%CI:84.6–99.3)	**24.2** (95%CI:14.6–37.0)	4.20 (95%CI:1.13–15.71)
HE staining + IHC	**47.3** (95%CI:37.0–57.9)	**94.1** (95%CI:69.2–99.7)	**97.8** (95%CI:86.8–99.9)	**24.6** (95%CI:15.1–37.1)	8.04 (95%CI:1.19–54.51)
HE staining + AFB	**30.1** (95%CI:21.3–40.6)	**82.4** (95%CI:55.8–95.3)	**90.3** (95%CI:73.1–97.5)	**17.7** (95%CI:10.4–28.3)	1.71 (95%CI:0.58–4.99)

### 3.3 Comparison of the distribution and overlap of positive results from the five tests

**Figure 5:**
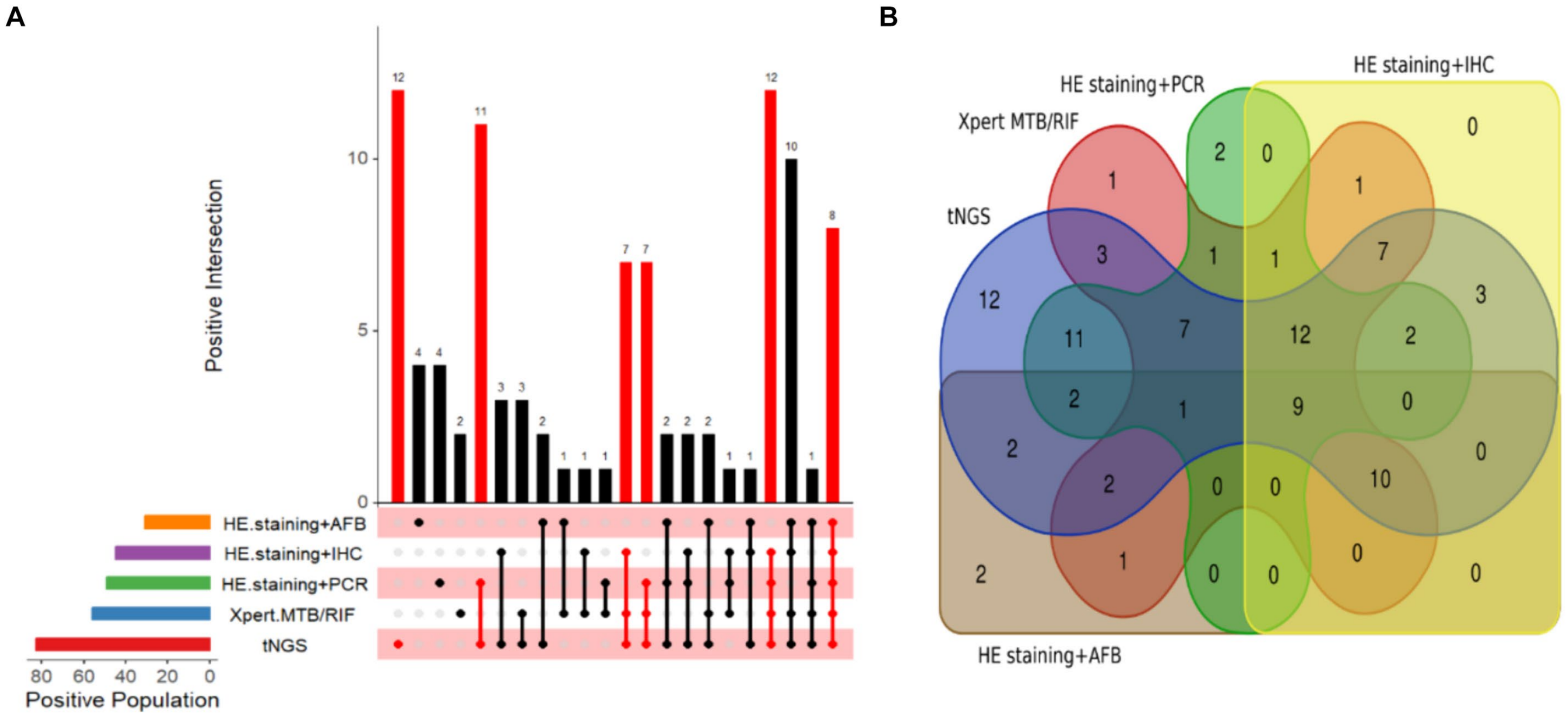
Visualization of TB detection test results. **(A)** Upset diagram of positive tests for the included patients. **(B)** Venn diagram of positive tests for TB patients.

In instances where other diagnostic methods yielded negative results, we found that tNGS alone identified 12 specimens as positive, whereas the number of positive specimens detected independently by HE staining combined with AFB, HE staining combined with PCR, Xpert MTB/RIF, and HE staining combined with IHC were 4, 4, 2, and 0, respectively (Figure 5A). Notably, tNGS achieved 100% PPV (12/12), successfully identifying five distinct forms of TB, four cases of PTB, four cases of cutaneous TB, two cases of lymph node TB, one case of tuberculous pleurisy, and one case of intestinal TB. By comparison, PPV for the Xpert MTB/RIF assay was only 50% (1/2), corresponding to one case of tuberculous pleurisy. PPV for HE staining combined with PCR was also only 50% (2/4), correctly identifying one case of lymph node TB and one case of skin TB. Similarly, PPV of HE staining combined with AFB was 50% (2/4), detecting one case of skin TB and one case of PTB. In addition, the abilities of tNGS and the other detection methods to identify TB patients were visualized using a Venn diagram (Figure 5B). The results revealed that tNGS shared 11 positive results with Xpert, seven positive results with Xpert and HE staining combined with PCR, 12 positive results with Xpert, HE staining combined with PCR, and HE staining combined with IHC, and nine positive results with all four of the other tests.

### 3.4 Comparison of pathogenic positivity rate between tNGS and other TB detection methods

For lung tissue samples, tNGS demonstrated statistically significant differences (*p* < 0.05) in pathogenic detection rate compared with three of the other detection methods, while differences between tNGS and Xpert MTB/RIF were not statistically significant (*p* = 0.128, Table 3). Regarding lymph node samples, significant disparities (*p* < 0.05) in pathogenic positivity rate were observed between tNGS and three of the other tests, with no significant differences detected (*p* = 0.172) between tNGS and the combination HE staining and PCR. Concerning skin tissue samples, a remarkable statistical difference (*p* < 0.05) was observed in pathogenic positivity rate between tNGS and all four of the other tests. All *p* values for tNGS were below the threshold of 0.05 (*p* < 0.001) when compared to the other four detection methods.

**Table 3:** Comparison of pathogenic positivity rate between tNGS and the other four TB detection methods.

Detection method	Lung		Lymph node		Skin		Overall	
	χ^2^	*p* value	Odds ratio	*p* value	Odds ratio	*p* value	χ^2^	*p* value
Xpert MTB/RIF	2.318	0.128	13.753	0.015	24.624	0.001	13.209	<0.001
HE staining + PCR	16.295	<0.001	6.444	0.172	7.933	0.041	21.813	<0.001
HE staining + IHC	9.805	0.002	22.240	0.002	49.465	<0.001	25.576	<0.001
HE staining + AFB	29.616	<0.001	37.975	<0.001	10.973	0.015	47.354	<0.001

### 3.5 Drug resistance results among 93 TB patients

The tNGS results revealed that variants conferred drug resistance in 10 of 93 (10.75%) TB patients. Specifically, seven patients were resistant to isoniazid (INH), with five cases attributed to mutations in the *katG* gene and two cases due to mutation in the *inhA* gene. Furthermore, six patients demonstrated resistance to RIF, with mutations occurring in the *rpoB* gene. By contrast, the GeneXpert MTB/RIF assay detected resistance to RIF with *rpoB* mutations in only two patients. Additionally, tNGS identified one patient with resistance to fluoroquinolone drugs, specifically due to mutations in the *gyrA* gene, and another patient who showed resistance to pyrazinamide drugs, with mutations in the *pncA* gene.

## 4 Discussion

Successful treatment of TB depends on widespread access to accurate diagnosis. In general, pathological examination is crucial for confident TB diagnosis, especially when smear and culture tests are negative, and in cases of EPTB (Chen et al., 2024). However, conventional detection methods are deemed inadequate to meet clinical needs due to delayed diagnosis and misdiagnosis (Fan and Xiang, 2021; Ma et al., 2021; Xin, 2021). Although some molecular detection methods such as Xpert can be of remarkable value in TB diagnosis, they perform poorly when assessing paucibacillary specimens (Theron et al., 2011; Ushio et al., 2016). Meanwhile, PCR has drawbacks including a high false positive rate and susceptibility to contamination from the surrounding environment (Guo Li-na et al., 2015). Notably, the applicability of these molecular methods is further limited because patients cannot provide qualified sputum samples, and EPTB patients have limited access to suitable clinical sample types (Chen et al., 2024). Luckily, numerous studies have demonstrated that tNGS is more effective than AFB-based, drug susceptibility testing (DST), and Xpert methods for diagnosing TB in sputum and BALF samples, and it can rapidly detect drug resistance (Daum et al., 2014; Chan et al., 2020; Yu et al., 2022; Liu et al., 2023). Nevertheless, the diagnostic value of tNGS for TB detection in pathological tissues remains unproven, despite its crucial importance in diagnosing EPTB. This is why we compared the diagnostic ability of tNGS for TB detection in pathological tissues with established approaches including HE staining, Ziehl-Neelsen staining, IHC with anti-MPT64, PCR, and Xpert MTB/RIF. We explored the potential clinical significance of tNGS in the diagnosis of TB using various pathological tissue samples.

We assessed the utility of tNGS using tissue samples based on true predictive ability, pathogenic positivity rate, and diagnostic accuracy. The results showed that tNGS achieved a sensitivity of 88.2% and a specificity of 94.1%, with its sensitivity significantly surpassing that of the other four tests. This suggests that tNGS possesses the benefit of exceptional sensitivity when it comes to identifying TB in tissues. IHC relies on the production of various polyclonal and monoclonal antibodies through tissue reactions. In particular, polyclonal antibodies possess the capacity to attach to various antigenic epitopes of a particular molecule, allowing tNGS to amplify the detection signal and hence the sensitivity when detecting MTB. Li-jing et al. (2014) discovered that the diagnostic efficacy of the polyclonal antibody IHC labeling technique for TB in pathological tissues was markedly superior to that of the acid-fast staining method (*p* < 0.005). This also confirmed why tNGS showed such exceptional sensitivity in our study. Nevertheless, tNGS did not exhibit a remarkable difference in specificity to the other four tests, possibly due to the limited sample size tested in this study, consequently leading to the introduction of statistical errors. Therefore, we integrated sensitivity and specificity to investigate the true predictive ability of tNGS for TB. The results revealed that tNGS surpassed the other four tests, as evidenced by its higher AUC (0.91).

In terms of pathogenic positivity rate, tNGS outperformed the other four testing methods. It consistently detected the highest number of positive cases (*n* = 12), encompassed the widest range of tuberculosis types (*n* = 5), and demonstrated the highest accuracy in terms of PPV (100%). If there was a need to rely on a single method when assessing TB puncture tissues, tNGS would be the optimal assay for TB detection. Additionally, when multiple methods were employed for the simultaneous identification of TB, tNGS always showed the highest overlap in positive detection compared with the other detection methods, indicating the reliability of tNGS in the joint detection of TB.

To delve deeper into the diagnostic performance of the most commonly used puncture tissues in clinical practice (lung, lymph node, and skin), we meticulously assessed the differences in pathogenic positivity rates between tNGS and the other four methods. Prior small-scale studies focusing on non-liquid specimens indicated that Xpert exhibits satisfactory performance in lung biopsy tissues (García-Basteiro et al., 2016), lymph node biopsy tissues (Dhasmana et al., 2014), and joint and bone biopsy tissues (Gu et al., 2015). However, from the CT values, it was considered that most of the tissues examined by Xpert are potentially specimens with high bacterial load (Hanrahan et al., 2014), which limits applicability in complex clinical environments. Thus, we inferred that the negligible discrepancy between tNGS and Xpert in lung puncture tissues (*p* = 0.128) in this study could be attributed to this reason. Lymph nodes are among the most susceptible sites for EPTB, providing niches for MTB growth and persistence (Ganchua et al., 2018). Reactivation of latent TB can initiate disease from lymph nodes and disseminate MTB to the lungs and various other organs (Ganchua et al., 2020). Additionally, the ability of a drug to reach the lymph nodes is typically significantly lower that its ability to penetrate into the blood, lung tissues, and lung granulomas, resulting in slow progress in drug treatment for lymph node TB (Das et al., 2020). Therefore, the prompt identification and effective management of lymph node TB dissemination are crucial. TB-PCR is a targeted nucleic acid amplification technique for *M. tuberculosis complex IS6110*. Das et al. (2020) employed TB-PCR to evaluate TB patients in low TB incidence areas, and discovered a discrepancy in sensitivity between smear-positive and smear-negative patients (97 vs. 79%). Furthermore, they suggested that despite the favorable performance of TB-PCR in detecting EPTB specimens with low bacterial load, its full potential remains untapped due to the significant false-positive rate resulting from technical characteristics and inadequate negative predictive values in regions with low incidence rates (Das et al., 2020). These findings for PCR are consistent with the detection characteristics observed in our current work (Figure 3).

Furthermore, EPTB poses significant challenges regarding the procurement of pathological tissue samples. For instance, organs such as lymph nodes may be situated in hard-to-reach areas or linked to vital organs, leading to an increased risk of trauma or surgical complexity during resection biopsy (the gold standard; Kiliçarslan et al., 2017). Additionally, the effectiveness of puncture biopsies is dependent on the proficiency and expertise of the operator, with the additional challenge of inadequate sampling impacting the accuracy of detection outcomes (Meng et al., 2022). Considering the aforementioned factors, although our study found no significant difference (*p* = 0.172) between tNGS and PCR for lymph node puncture samples, we remain convinced that PCR-based methods will always struggle to fulfill the clinical requirements for analyzing such pathological tissues. Importantly, obtaining lymph node pathological tissues for clinical diagnosis is not as convenient as obtaining skin samples for detecting skin TB, and inaccurate test results can potentially inflict physical and psychological distress upon patients.

Resistance to anti-TB drugs is a major challenge in the treatment of TB. Recently, a multicenter study used tNGS to evaluate clinical availability for the diagnosis of drug-resistant TB (DR-TB) (Liu et al., 2024). The results showed a remarkable concordance rate of 99.44% (179/180) with the culture assay for identifying the MTB complex. The sensitivity of NanoTNGS for detecting drug resistance was 93.53% for RIF, 89.72% for INH, 85.45% for ethambutol (EMB), 74.00% for streptomycin, and 88.89% for fluoroquinolones. The sensitivity of NanoTNGS for RIF-resistant TB was 9.73% higher than that of Xpert MTB/RIF. Although this study did not conduct phenotypic drug sensitivity testing on punctured tissues, it is known that tNGS can simultaneously detect MTB and assess the sensitivity of >10 anti-TB drugs. Within our study, we employed tNGS to identify resistance to all first-line anti-TB drugs, namely RIF, INH, EMB, and pyrazine (PZA), in pathological tissues. tNGS was particularly effective in detecting PZA resistance, which was challenging to uncover through phenotype drug sensitivity testing (pDST) (Diacon et al., 2012; Zhang et al., 2014). This is because tNGS can selectively amplify drug resistance-related regions of the MTB complex (MTBC) genome, and rapidly provide results directly from clinical specimens, offering higher sensitivity than other detection methods. In conclusion, tNGS offers a hopeful substitute for pDST in high-burden environments. For tissues, ongoing research into drug resistance detection is warranted.

Our current research has some limitations. Firstly, this study was conducted retrospectively, making encountering patient selection bias inevitable. Secondly, considering the diagnostic accuracy of various detection methods, the patients recruited in this study might have a relatively high bacterial load, which prevented us from highlighting the superiority of tNGS in detecting EPTB and specimens with low bacterial load. Thirdly, the absence of phenotypic drug sensitivity testing might undermine the persuasiveness of our resistance results obtained through tNGS. Lastly, there is the possibility of biases in tests conducted by different organizations and personnel, which could potentially impact the applicability of experimental findings.

Our tNGS assay proved to be highly sensitive for detecting TB in tissues, even more so than four established methods, and it has great potential for rapidly defining drug resistance elements. Additionally, we propose that a combination of multiplex PCR and tNGS can be a highly efficacious strategy for identifying EPTB and populations with low bacterial loads. Multiplex PCR can be employed to selectively amplify various antigenic epitopes and resistance sites of MTB. By leveraging the long-read sequencing capabilities of tNGS, challenging and low-load pathogens could be detected, and virulence and resistance genes could be identified.

## 5 Conclusion

The tNGS assay was more sensitive than four established diagnostic methods for rapidly diagnosing TB in tissues. The assay also has considerable potential for rapidly pinpointing drug resistance elements. Improving the sensitivity of resistance detection, reducing costs, and improving bioinformatics data analysis and integration could further enhance the clinical applicability of this powerful TB diagnostic tool.

## Data availability statement

The datasets presented in this study can be found in online repositories. The names of the repository/repositories and accession number(s) can be found here: NCBI BioProject (https://www.ncbi.nlm.nih.gov/bioproject), accession number: PRJNA1127786.

## Ethics statement

The studies involving humans were approved by Nanjing Second Hospital Medical Ethics Committee. The studies were conducted in accordance with the local legislation and institutional requirements. The participants provided their written informed consent to participate in this study.

## Author contributions

WG: Data curation, Investigation, Validation, Writing – original draft, Writing – review & editing. CY: Data curation, Formal analysis, Methodology, Resources, Writing – original draft. TW: Data curation, Investigation, Methodology, Writing – original draft. YG: Data curation, Validation, Writing – review & editing. YZ: Writing – original draft.
